# The 20 item prosopagnosia index (PI20): relationship with the Glasgow face-matching test

**DOI:** 10.1098/rsos.150305

**Published:** 2015-11-04

**Authors:** Punit Shah, Sophie Sowden, Anne Gaule, Caroline Catmur, Geoffrey Bird

**Affiliations:** 1 MRC Social, Genetic and Developmental Psychiatry Centre, Institute of Psychiatry, Psychology and Neuroscience, King’s College London, London, UK; 2 Department of Psychology, Institute of Psychiatry, Psychology and Neuroscience, King’s College London, University of London, London, UK; 3 Department of Psychological Sciences, Birkbeck College, London, UK; 4 Institute of Neurology, University College London, London, UK; 5 Institute of Cognitive Neuroscience, University College London, London, UK; 6 Department of Psychology, University of Surrey, Surrey, UK

**Keywords:** developmental prosopagnosia, self-report, Glasgow face-matching test, face matching, prosopagnosia, face perception

## Abstract

The 20 item prosopagnosia index (PI20) was recently developed to identify individuals with developmental prosopagnosia. While the PI20’s principal purpose is to aid researchers and clinicians, it was suggested that it may serve as a useful screening tool to identify people with face recognition difficulties in applied settings where face matching is a critical part of their occupation. Although the PI20 has been validated using behavioural measures of face *recognition*, it has yet to be validated against a measure of face-*matching* ability that is more representative of applied settings. In this study, the PI20 was therefore administered with the Glasgow face-matching test (GFMT). A strong correlation was observed between PI20 and GFMT scores, providing further validation for the PI20, indicating that it is likely to be of value in applied settings.

## 1. Introduction

Face recognition is critical for social interaction, and has therefore been the subject of intensive scientific investigation. Studying individuals who have a selective impairment in face recognition in the absence of brain injury—those with developmental prosopagnosia (DP)—has contributed to understanding the mechanisms underlying typical face recognition (see [1]). Less attention, however, has been directed to studying DP within the context of individual differences in face recognition ability and the practical implications of this condition on wider society. This is partly because identifying DP and quantification of its severity has not been standardized in the way in which it has for other developmental disorders (e.g. autism—autism diagnostic observation schedule; [2]). This lack of standardization is an obstacle to the development of formal diagnostic criteria, and until DP is better recognized among clinicians it is less likely to be considered in non-clinical settings [3].

To begin addressing these issues, the 20 item prosopagnosia index (PI20 [4]) was developed; a self-report measure designed to help identify adults with DP and, more generally, to quantify face recognition difficulties in the general population. The PI20’s psychometric properties, as previously reported, are good, and it has good construct validity. It is therefore able to successfully distinguish individuals known to have DP from typical individuals. Furthermore, scores on the PI20 predict face recognition (e.g. Cambridge face memory test, CFMT [5]) abilities, but are not related to performance on the Cambridge car memory test [6]. This pattern of results suggests that the PI20 indexes face recognition ability specifically, rather than providing a measure of general object recognition ability. The reliability and validity of the PI20 suggest that it may benefit researchers interested in face perception, as well as clinicians who encounter DP or co-occurring DP in their practice. In a research context, the PI20 will be useful for classifying people with DP for inclusion in research samples and to exclude such individuals from studies of typical face perception. In clinical contexts, the PI20 may prove to be particularly useful during differential diagnosis in tertiary psychiatric units where co-occurring prosopagnosia is most likely to be reported [7].

The PI20 can be administered quickly—without specialist equipment or training—but its potential for use in applied settings is currently unknown. Although validated against the CFMT, widely regarded as the ‘gold-standard’ measure of face recognition ability within DP research, the CFMT may not measure the type of face processing skills required by, for example, passport control officials and police officers. First, the CFMT has been argued to conflate measurement of face memory and face recognition [8–10]. Second, the test format is—by design—not reflective of real-world situations; it includes only male faces from which hairlines have been cropped. Third, it is not representative of occupational settings where face *matching* is required, such as comparing two images taken on different cameras (e.g. photo-to-photo matching from surveillance footage and police booking photographs) or the photo-to-person matching required at passport control, for example.

In recognition of the foregoing issues (see also [11–14]), Burton *et al.* devised the Glasgow face-matching test (GFMT [9]); a psychometric instrument designed for applied face-matching research, with a view to using the measure in security settings (see Methods for detailed information). The GFMT has been used in several applied studies (see [15]), for example with passport control officers [16]. To investigate whether the PI20 is suitable for use in applied settings, this study, therefore, sought to investigate the relationship between scores on the PI20 and performance on the GFMT.

## 2. Method

### 2.1 Participants

One hundred and ninety adults (59 males; 15 left-handed) aged between 18 and 75 years (*M*_age_=27.72 years, s.d.=10.66) participated in the study. The sample size was chosen to match that used by Burton *et al.* [9] when they constructed the GFMT. In addition, power analysis [17] indicated that this sample size would be sufficient to detect a moderate sized correlation (where *r*=0.30, power=0.95, *α*=0.05) between the GFMT and PI20. Participants only reported their age, sex, handedness and all had normal or corrected-to-normal vision. Participants were recruited using a local participant database and gave informed consent prior to their participation. Ethical clearance was granted by the local ethics committee and this study will be reported in line with recommendations from Simmons *et al.* [18].

### 2.2 Materials and procedure

The PI20 [4] (see the electronic supplementary material) required participants to indicate the extent to which 20 statements described their face recognition difficulties on a five-point Likert scale (strongly agree to strongly disagree), from which a score between 20 (no face recognition difficulties) and 100 (severe face recognition difficulties) was derived.

**Figure 1.**
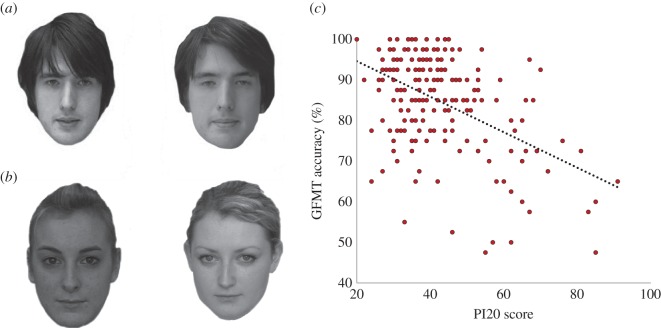
Examples of (*a*) same and (*b*) different-face trials from the short version of the Glasgow face-matching test (GFMT [9]). The GFMT is freely available from http://www.facevar.com/downloads and should be credited to Burton *et al.* [9] if it is used in published research. People who appear in the GFMT gave written consent for their images to be used in the context of academic research and publication. (*b*) The simple correlation between 20-item prosopagnosia index (PI20 [4]) scores and performance on the GFMT (*r*=−0.49, *p*<0.001).

The abbreviated version of the GFMT [9] was used. This comprised 40 pairs of faces. All faces were of neutral expression and comprised high-resolution (1000×700) greyscale images. To reflect real-world face matching, hairlines were intact, 40% of face pairs were female, and photos within each pair were taken using two different cameras. Of the 40 test pairs, 20 are same-face trials, in which two images of the same person are presented alongside one another (figure 1*a*). The other 20 pairs comprised different-face trials (figure 1*b*) of people that were similar in appearance. The two images are presented simultaneously in order to eliminate memory demands and participants were required to indicate whether the two images were the same or different. In accord with Burton *et al.*’s instructions, the GFMT was administered on a computer screen at 100% scale and self-paced, which took participants approximately 7 min (*M*=7.6 min, s.d.=3.0 min) to complete. The order of administration of the PI20 and GFMT was counterbalanced across participants.

## 3. Results

The mean PI20 score (*M*=41.99, s.d.=13.40) was consistent with the original validation study [4], the PI20’s internal consistency (Chronbach’s *α*=0.93; Guttman split-half correlation=0.91) was good, and its unifactorial structure was found to be robust. Performance on the GFMT ranged between 47.5% and 100.0% (*M*=85.00, s.d.=11.92). Signal detection analysis [19] was conducted to measure discrimination performance (*d*′) independent of response bias. Reflecting percentage accuracy, *d*′ scores ranged between −0.25 and 3.29 (*M*=2.27, s.d.=0.85).

PI20 scores correlated significantly with percentage accuracy (*r*=0.49, *p*<0.001; figure 1*c*) and *d*′scores (*r*=0.47, *p*<0.001) on the GFMT. To control for the influence of demographic factors and time taken to complete the GFMT on face-matching performance, participant age (years), sex (1, male; 2, female) and completion time (seconds) were entered into the first step of a hierarchical regression, with PI20 scores entered second. Participant age (*β*=−0.14, *p*=0.051), sex (*β*=0.21, *p*=0.004) and completion time (*β*=0.23, *p*=0.001) were all predictors of GFMT performance, together accounting for 12.8% of the variance. Female participants outperformed (*M*=87.08, s.d.=10.92) male participants (*M*=80.38, s.d.=12.81, *t*_188_=3.70, *p*<0.001, *d*=0.56), whereas older age was weakly associated with less accurate GFMT performance (*r*=−0.15, *p*=0.040). Importantly, however, the PI20 score remained the strongest predictor (*β*=−0.45, *p*<0.001), accounting for a further 19.2% of unique variance. This pattern of significance was identical when *d*′ scores were entered into analyses as the measure of GFMT performance.

## 4. Discussion

The PI20 was recently designed to support research and clinical practice involving DP, which guided its validation using well-established measures of face *recognition* ability. Because the PI20 also holds potential for use in applied settings, we sought to examine the relationship between the PI20 and the GFMT; a psychometric tool designed to measure face-*matching* ability in applied settings.

Following previous work [4], this paper demonstrates that the PI20 is a robust self-report measure as its psychometric properties hold when administered to a large sample of new participants. In addition, this study supports the claim that individuals have insights into their face perception ability. The literature investigating this question has produced equivocal findings to date, with some studies reporting correlations between confidence judgements and face-matching performance and others reporting no relationship [20–22]. It is possible that previous findings of a lack of a correlation between confidence and accuracy are due to the use of single-item questions designed to be answered with respect to a single time-point. These measures may produce noisier estimates of confidence than those obtained through administration of the PI20, whereby estimates are derived from responses to multiple questions, and participants are given the opportunity to estimate their average confidence based on a longer time period (i.e. their lifetime). It is also possible that within-task measures of confidence prompt participants to estimate their confidence on a given trial with respect to their own average performance, rather than with respect to other individuals as measured by the PI20, and that participants are able to do the latter but not the former. Both these possibilities are worthy of further investigation.

There was substantial inter-individual variation in GFMT performance, in line with that found by Burton *et al.* [9,16], supporting the suggestion that such tests should be administered when selecting personnel for occupations requiring face matching. As has been previously observed [23], including recently using a modified version of the GFMT [24], females outperformed males. The size of the sex effect was, however, small, particularly when compared with the large individual differences observed in face-matching performance in the sample as a whole. The relationship between age and GFMT performance was also small, though marginally significant, in line with a recent study on age-related decline in unfamiliar face matching [25]. Most importantly, with respect to the aim of this study was the finding that the PI20 was the strongest predictor of face-matching performance over and above that accounted for by demographic variables. Because a strong relationship was observed between PI20 score and performance on the GFMT, this provides further construct validation for the PI20 and indicates its potential for use in applied settings.

### 4.1 Outstanding issues, practical limitations and future directions

The majority of items on the PI20 enquire about *familiar* face recognition, yet scores on this measure predict performance on the GFMT, a measure of *unfamiliar* face matching. This is of interest owing to ongoing debate about the degree to which processing of familiar and unfamiliar faces recruit common or distinct neurocognitive mechanisms [26]. Behavioural research on this topic provides strong evidence that (upright) familiar faces recruit processes which are not recruited for unfamiliar faces (see [27] for an excellent overview of this evidence), although this research also demonstrates that familiar and unfamiliar faces may recruit some common, face-specific, processes [28]. Investigation of the neural correlates of face processing has provided mixed evidence for a dissociation between the processing of familiar and unfamiliar faces however, with many studies finding overlapping neural correlates of familiar and unfamiliar face processing [27,29]. The difference in the *magnitude* of the correlations between the PI20 and various objective measures of face perception—in this study and those reported in Shah *et al*. [4]—support the theoretical distinction between familiar and unfamiliar faces. As the PI20 focuses on recognition of familiar faces, PI20 scores show a greater (*Z*=2.44, *p*=0.015) correlation with scores on the famous face recognition test (*r*=−0.813) than with scores on the CFMT (*r*=−0.683), which assesses recognition of recently viewed novel faces. With respect to separable recognition and matching processes, the correlation between the PI20 and unfamiliar face matching (GFMT performance) is significantly weaker than that between PI20 scores and CFMT performance, *Z*=−2.45, *p*=0.014, and famous face recognition, *Z*=−5.65, *p*<0.001. While this pattern of results lends some support to the distinction between familiar and unfamiliar face perception, and between recognition and matching, it should be noted that this study was not designed to address this question and that comparisons are made across distinct participant samples.

Three factors should be considered before the use of an instrument such as the PI20 in applied settings. The first is that, in order to gain or retain employment, it is possible that individuals may complete self-report measures in a manner which they believe will enhance their prospects, rather than accurately reflecting their true ability. Second, the distribution of PI20 scores was—in accordance with Shah *et al*. [4]—skewed towards lower scores (figure 1*c*), reflective of low prosopagnosic traits in the population. The PI20 is therefore particularly sensitive at identifying individuals with DP, but relatively less able to identify individuals with exceptionally good face-matching ability—for which objective measures of face matching are more appropriate. Third, although the PI20 has good predictive validity at the group level, a non-trivial number of participants report good face processing ability on the PI20 yet perform poorly on the GFMT. This highlights the worrying tendency for some individuals to substantially overestimate their face-matching ability, which could, for example, have important implications in legal settings. The PI20 will therefore enable future research into why individuals under- or overestimate their face-matching ability, perhaps in relation to individual differences in personality (see [30]).

In the light of the above-mentioned issues, we suggest that the PI20 should *not* be considered as an alternative to objective measures of face-matching ability; rather it is hoped the PI20 will be used as a complementary instrument. Wherever possible, the PI20 should be administered with behavioural measures (e.g. GFMT) to form a detailed perceptual profile of an individual. This is important, because many factors—such as misinterpretation of instructions or test anxiety—may lead someone to perform badly on behavioural measures of face matching. This is particularly likely when they are not administered by trained experimenters in applied settings. Where PI20 and behavioural test scores converge, it is likely that users (e.g. employers) can be more confident of their validity. A direct test of the combined predictive power of the PI20 and objective face perception scores on ecologically valid face-matching tasks (e.g. photo-to-person tests [16]) is therefore a priority for future research.

## 5. Conclusion

In summary, this study indicates that the PI20 holds potential for use in applied settings, particularly when combined with an objective test of face matching such as the GFMT. It is hoped that research leading to the development of the PI20 will be extended by occupational and forensic research groups, using participants recruited within applied settings, and in field research involving photo-to-person matching.

## Supplementary Material

PI20 Questionnaire: A printable version of the

## Supplementary Material

PI20 for download and use in research Dataset: A file containing the data collected during the study

## Data accessibility

The dataset supporting this article is available as electronic supplementary materials.
